# Cracks and lights of assessment implementation in competency‐based medical education

**DOI:** 10.1111/medu.70213

**Published:** 2026-04-10

**Authors:** Valdes Roberto Bollela, Ugo Caramori

**Affiliations:** ^1^ Ribeirão Preto School of Medicine University of São Paulo Ribeirao Preto Brazil; ^2^ School of Medical Sciences University of Campinas (UNICAMP) Campinas Brazil

## Abstract

This Commentary sheds light on how cracks in #CBME can be used as opportunities for guidance, shared burdens and restored meaning. There can be no learning‐centred education without learners and their experiences; assessment must be designed to make those visible, interpretable and protected. #MedEd

*There is a crack, a crack in everything. That's how the light gets in*. Leonard Cohen (1992), *Anthem* .

Competency‐based medical education (CBME) challenges long‐standing assumptions regarding training, competence, and accountability. At its best, CBME aligns curriculum, assessment, learning and progression with the realities of clinical work and societal expectations for trustworthy professionals. The central claim of CBME is integration. Assessment is not an add‐on but part of the educational architecture that shapes what is learned, valued and rewarded.^1^, ^2^

Assessment is not an add‐on but part of the educational architecture that shapes what is learned, valued and rewarded.^1^, ^2^

As CBME scales, predictable frictions arise in heterogeneous clinical environments and may lead to cracks between the intended design and the lived experiences of trainees and physicians. Such cracks should not be considered evidence of failure or cause for retreat but rather an opportunity to see through them and catalyse continuous dialogue, evaluation and improvement.

In this commentary, we reflect on an experience arising from a large‐scale national implementation of competency‐based education in postgraduate training, which offers a timely illustration. In a qualitative case study of paediatric residency in the inaugural cohort of *Competence by Design* (CBD), residents supported CBD's stated purpose but described implementation as shifting responsibility and administrative burden onto learners, generating variable feedback, functioning more as assessment of learning than assessment for learning and revealing difficulty among faculty in embracing the system.^3^ This pattern is consistent with broader concerns about assessment burden and unintended incentives in CBME.^4^, ^5^

A central risk of these studies lies in conflating the CBME paradigm with the assessment architecture built around it and so, blaming CBME for problems that are better understood as consequences of design choices, resource availability, and local institutional and cultural norms. To address these cracks productively, the design, scale and placement of assessment systems within CBME should be scrutinised. Learner‐centredness does not imply convenience, reduced expectations or a learner‐protective mechanism that eliminates discomfort and effort. It assumes active engagement in learning, iterative self‐assessment, timely feedback and flexible developmental pathways. Cognitive load, reflective work and sustained clinical engagement are not deviations from learner‐centeredness; rather, they are conditions of professional growth.

A central risk of these studies lies in conflating the CBME paradigm with the assessment architecture built around it.

The problem emerges when progression becomes primarily dependent on documentation rather than meaningful assessment practices. When assessment is reduced to binary thresholds—achieved/not achieved, completed/not completed—it becomes an accumulation system where learners will respond adaptively: They ‘collect’ entrustable professional activities (EPAs), cultivate strategies to optimise passing (including seeking out low‐risk assessors) and devote attention to dashboards, targets and spreadsheets. When progression depends on documentation, EPAs become currency, and dashboards become the hidden curriculum that drives gaming.

When progression depends on documentation, EPAs become currency, and dashboards become the hidden curriculum that drives gaming.

CBME implementations may also be vulnerable to *milestoneisation*: the steady multiplication of auditable checkpoints justified in the name of standardisation and defensible decision‐making.^6^ Such architectures can displace the original purpose of EPAs—to ground judgement in developmental entrustment decisions linked to authentic clinical work—by privileging threshold attainment. *Milestoneisation* trades developmental judgement for auditable checkpoints, moving assessment from learning to certification. Over time, learners may avoid documenting struggle or novelty because low ratings can carry farther than formative guidance, especially when the stakes feel unclear or high.^7^

*Milestoneisation* trades developmental judgement for auditable checkpoints, moving assessment from learning to certification.

Three design elements are foundational when building an assessment for learning system. First, learners require protected time to reflect on their experiences—work that is not easily counted but is essential for professional identity development. Second, interpretive responsibility must be explicit: A competence committee should synthesise the meaning of assessment information (including narrative data and mentor perspectives) rather than simply tallying outputs. Third, learners need integrated, timely feedback to recalibrate their goals and strategies along the way. Learners' engagement is essential in programmatic assessment and may help to design and refine transparent processes (rules, remediation, and appeals). Final high‐stakes decisions remain with faculty. Assessment literacy across the institution is essential so learners can contribute to assessment, not just being assessed.^7^, ^8^, ^9^

When such safeguards are absent, a market‐like relationship with assessment may arise, with accumulation, exchange and compliance dominating. In this climate, the neoliberal features often attributed to CBME are less about CBME itself and more about institutional cultures that normalise surveillance, individualise responsibility for system failures and reward what is measurable over what is meaningful. Ironically, an apparatus designed to assure quality can end up eroding the learning environment that CBME was meant to support.

Importantly, the meaning of these dynamics shifts according to context and positionality. In resource‐rich, highly regulated systems, particularly in the Global North, frictions are often experienced as administrative and technological. In different settings, CBME can function differently as a challenge to opaque curricula, implicit hierarchies and historically unaccountable authority structures. When properly positioned, CBME can be emancipatory, clarifying expectations, redistributing curricular authority and reframing medical education as a public good at the frontlines of care. In these contexts, an excess of assessment moments (summative and/or formative) is not only burdensome but also unrealistic and unworkable.

The broader issue is not that CBME demands too much from learners. The issue is misalignment: assessment systems that overvalue what can be efficiently captured compared to what must be cultivated—judgement, professionalism, collaboration, adaptability and identity formation. If we do not name this misalignment, critiques will continue to target CBME as a paradigm rather than the assessment designs, insufficient faculty development and organisational conditions that have made such deviations possible.

Repairing the cracks without sealing out the light requires a disciplined return to programmatic assessment.^1^, ^2^ This means combining multiple low‐stakes observations across contexts; privileging narrative, dialogue and workplace meaning; and relying on collective professional judgement for progression decisions. This means designing digital platforms as integrators (a learner‐owned portfolio that supports reflection and synthesis) rather than as fragments that incentivise task completion. It also means supporting faculty through practical development in observation, narrative feedback, calibration and shared accountability for assessment work.^4^, ^5^

Implementation is also a challenge. Change management shapes what is normalised. When people work together in groups, they predictably move through phases of forming, storming, norming, and performing. The storming phase—when workload rises, morale drops and resistance becomes visible—is not failure but a high‐yield learning moment for adjustment.^10^ When early friction is mistaken for learner deficiency or faculty non‐compliance, organisations miss the chance to understand the cracks that friction reveals and to see through them. Sealing the cracks in CBME instead of examining them diminishes the light of guidance, shared burdens and restored meaning that they could provide.

Sealing the cracks in CBME instead of examining them diminishes the light of guidance, shared burdens and restored meaning that they could provide.

There can be no learning‐centred education without learners and their experiences; assessment must be designed to make those visible, interpretable and protected within a development‐oriented and shared educational environment.

## AUTHOR CONTRIBUTIONS

**Valdes Roberto Bollela:** Conceptualization; writing—original draft; writing—review and editing; validation. **Ugo Caramori:** Conceptualization; writing—original draft; writing—review and editing; validation.

## Data Availability

Data sharing is not applicable to this article as no datasets were generated or analysed during the current study.
